# Pharmacological Preconditioning by Adenosine A2a Receptor Stimulation: Features of the Protected Liver Cell Phenotype

**DOI:** 10.1155/2015/286746

**Published:** 2015-10-11

**Authors:** Elisa Alchera, Chiara Imarisio, Giorgia Mandili, Simone Merlin, Bangalore R. Chandrashekar, Francesco Novelli, Antonia Follenzi, Rita Carini

**Affiliations:** ^1^Department of Health Science, University of Piemonte Orientale, 28100 Novara, Italy; ^2^Centre for Experimental and Clinical Studies (CERMS), Azienda Universitaria Ospedaliera Città della Salute e della Scienza Città di Torino, 10100 Turin, Italy; ^3^Department of Molecular Biotechnology and Heath Sciences, University of Turin, 10100 Turin, Italy

## Abstract

Ischemic preconditioning (IP) of the liver by a brief interruption of the blood flow protects the damage induced by a subsequent ischemia/reperfusion (I/R) preventing parenchymal and nonparenchymal liver cell damage. The discovery of IP has shown the existence of intrinsic systems of cytoprotection whose activation can stave off the progression of irreversible tissue damage. Deciphering the molecular mediators that underlie the cytoprotective effects of preconditioning can pave the way to important therapeutic possibilities. Pharmacological activation of critical mediators of IP would be expected to emulate or even to intensify its salubrious effects. *In vitro* and *in vivo* studies have demonstrated the role of the adenosine A2a receptor (A2aR) as a trigger of liver IP. This review will provide insight into the phenotypic changes that underline the resistance to death of liver cells preconditioned by pharmacological activation of A2aR and their implications to develop innovative strategies against liver IR damage.

## 1. Ischemia/Reperfusion Injury of the Liver

Inflow occlusion during liver surgery with consequent reperfusion causes ischemia/reperfusion (I/R) injury of the liver. I/R injury is recognised as a main risk factor after major hepatic surgery and liver transplantation since it may affect patients recovery and carries a risk of poor postoperative outcome [1].

Hepatic I/R injury is a complex, multifaceted process that occurs during the ischemic period as well as during the reperfusion phase. During ischemia, mitochondrial deenergization, ATP depletion, and ionic and volume alterations lead to liver cell necrosis. Upon oxygen readmission, reactive oxygen species (ROS) production by uncoupled mitochondria promotes oxidative stress and mitochondrial permeability transition and is associated with a decreased capacity to synthesize ATP. Caspase activation, necrosis, and apoptosis of liver cells and activation of the inflammatory reactions follow these events. Resident Kupffer cells and infiltrating neutrophils and lymphocytes release ROS, proteases, and cytokines and further contribute to the progression of hepatic injury [2–4]. Preclinical studies have shown several strategies able to reduce hepatic damage by individually targeting the different alterations that contribute to I/R injury [2–6]. Their potential adverse effects and their focused approach have inhibited, however, their translation to patients and, to date, no definitive methods have become part of the clinical practise [1, 2].

## 2. Hepatic Ischemic Preconditioning

The term ischemic preconditioning (IP) refers to the increase in tissue tolerance to ischemia/reperfusion (I/R) damage that can be induced by the preexposure to brief periods of ischemia followed by reperfusion [7]. This phenomenon was first described by Murry et al. in the myocardium [8], but it was subsequently observed in many other tissues [7]. In liver, studies in rodents have shown that 10 min interruption of blood flow followed by 10 min reperfusion reduces hepatic injury, oxidative stress, microvascular disturbances, and inflammation during a subsequent extended period of I/R [1–8].

The demonstration of the pleiotropic protective effects of IP in the experimental models has raised hopes that it could be a useful and easy technique to reduce I/R injury in human liver surgery. IP surgical application, however, has the disadvantage of inducing trauma to major vessels and stress to the target organ [9]. Moreover the contrasting outcomes of the first clinical studies, the different protocols of IP application in humans, and the variable clinical settings have not allowed a definitive demonstration of the benefit of the clinical application of IP [9–13].

This observation has inhibited, by now, the routine use of IP in human liver surgery and has indicated the need of more efficient approaches to activate IP in patients. In this regard, the pharmacological induction of liver preconditioning by targeted activation of one or more of the critical molecular mediators of IP may represent a more reliable technique to activate the intrinsic system of hepatoprotection in patients.

## 3. Adenosine A2a Receptor Activation: A Main Trigger of Hepatic Preconditioning

The nearly 25 years' research on liver IP has demonstrated that its applications induce deep modifications of liver tissue that make liver cells resistant to damage. The knowledge of the molecular changes responsible for the production of such protected liver cell phenotypes is however still incomplete. To date one of the established notions is the role of the adenosine A2a receptor (A2aR) activation as an inductor of liver preconditioning. Adenosine mainly originates by the breakdown of adenine nucleotides and even a transient damage of cell membranes, like that induced by the brief ischemic stress of IP, leads to massive ATP increase in extracellular space with rapid formation of adenosine [14]. Since the early reports of Peralta et al. [15, 16],* in vivo* and* in vitro* studies have shown that IP increases extracellular adenosine levels that in turn triggers IP protective effects upon stimulation of A2aR of liver cells. Consistently pretreatment with adenosine A2 receptor agonists enhances liver tolerance against hypoxia and I/R damage, while pharmacological or genetic inhibition of A2aR activation prevents the beneficial effects of IP [15–25].

The mechanisms responsible for A2aR-mediated hepatoprotection during IP are both indirect and direct. The indirect mechanisms depend on the maintenance of nitric oxide (NO) synthesis [15, 16] induced by preventing the downregulation of NO synthase of liver endothelial cells induced by I/R [26]. The direct effects are due to the activation of intracellular survival pathways as a consequence of the stimulation of the A2aR expressed on liver cells.

## 4. Adenosine A2a Receptor Activation Protects Hepatocyte Hypoxic Damage

**Figure 1:**
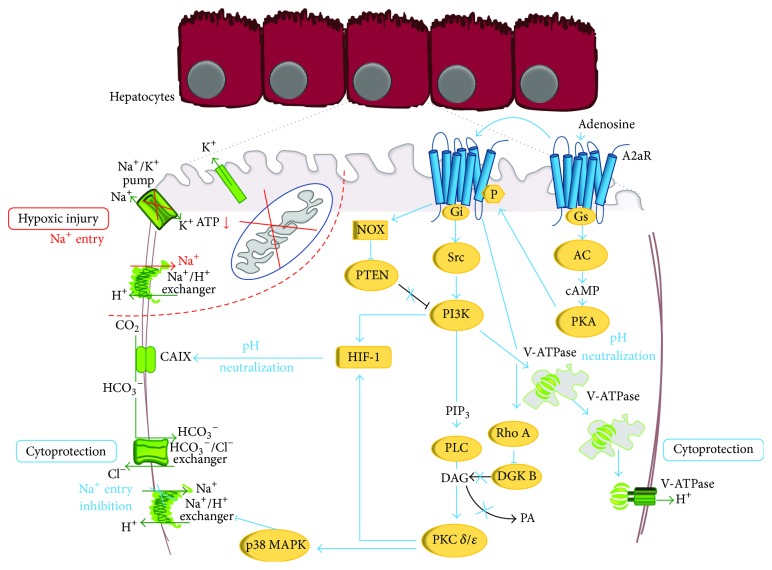
Molecular mechanisms involved in hypoxic injury of primary rat hepatocytes and in their protection upon A2aR stimulation. Hypoxic damage: ATP depletion causes intracellular acidosis, inhibition of the Na^+^/K^+^ ATPase, and activation of the Na^+^/H^+^ exchanger with an increase in intracellular Na^+^ content and activation of the K^+^ channel. A2aR protection: A2aR stimulation induces the sequential activation of PKA, Gs and Gi protein, Src, PI3K, PLC, PKC *δ*, and *ε* and p38 MAPK. A2aR also inhibits the negative regulators of PKC and PI3K, DGK, and PTEN. PI3K activates V-ATPase that maintains intracellular pH avoiding the activation of the Na^+^/H^+^ exchanger and Na^+^ overload. PI3K and PKC *δ* and *ε* activate HIF with production of CAIX. CAIX converts CO_2_ into bicarbonate that enters into hepatocyte through the Cl^−^/HCO_3_^−^ exchanger. This neutralizes intracellular pH without activation of the Na^+^/H^+^ exchanger and the consequent Na^+^ increase. (See also text and [19, 20, 27, 28, 36, 37, 40].)

In the past years, we have employed the* in vitro* model of primary rat hepatocytes preconditioned with a brief hypoxia-reoxygenation period to investigate the intracellular mechanisms responsible for the direct hepatoprotective action of A2aR stimulation. These studies have shown that A2aR stimulation activates a complex array of protective signals that contribute to the induction of hepatocytes resistance to hypoxic damage (Figure 1). Upon A2aR stimulation, with adenosine or pharmacological agonists, the activation of Gs protein and consequently of adenylate cyclase and protein kinase A (PKA) occurs [19, 20, 27]. PKA phosphorylates A2aR and shifts its coupling to Gi protein and Src kinase thus activating the surviving mediator phosphatidylinositol-3-kinase (PI3K) and its downstream effector Akt [21]. This allows the stimulation of phospholipase C, the recruitment of the specific isoforms *δ* and *ε* of protein kinase C (PKC), and the activation of p38 MAPK [19, 20, 27]. Full activation of preconditioning responses also needs downmodulation of inhibitory enzymes of PKC and PI3K. Hypoxic preconditioning as well as A2aR stimulation induces, in fact, a RhoA-GTPase-dependent inhibition of the diacylglycerol kinases *θ*, thus increasing diacylglycerol (DAG) and sustaining activation of the DAG-dependent PKC *δ* and *ε* [28]. Consistently recent “*in vivo*” studies with specific PKC *δ* inhibitors confirmed the critical role of PKC and, particularly, of PKC *δ* in mediating the protective effect of IP [25]. A2aR stimulation also induces the degradation of the PI3K inhibitor, phosphatase and tensin homologue deleted from chromosome 10 (PTEN), through a NADPH oxidase-dependent mechanism, thus allowing the maintenance of the PI3K-dependent signals [29]. The above observations indicate a key role played by PI3K and p38 MAPK in hepatocyte preconditioning as also confirmed by* in vivo* studies that reported a marked increase in the dual phosphorylation of hepatic p38 MAPK [30] and demonstrated the implication of PI3K in mediating hepatoprotection in preconditioned liver [31].

Biochemical studies shed light on mechanisms by which these protective signal networks induce the increased resistance of preconditioned hepatocytes to hypoxic injury.

As illustrated in Figure 1, hepatocytes death upon ATP depletion is precipitated by the deregulation of Na^+^ homeostasis [32]. An irreversible increase of intracellular Na^+^ promotes, in fact, hepatocytes killing induced by several insults including oxidative stress, mitochondrial toxins, and warm and cold hypoxia and at the first phases of reoxygenation [32–35]. Na^+^ alterations that follow ATP depletion are the result of a combined block of the ATP-dependent Na^+^ efflux through the Na^+^/K^+^ ATPase and of the activation of Na^+^/H^+^ exchanger and Na^+^/HCO_3_^−^ cotransporter in response to cytosolic acidification [32]. In the metastable phase that precedes death, hepatocytes respond to the progressive increase of intracellular Na^+^ with the stimulation of the volume regulatory decrease mechanisms, that is, activation of the K^+^ channels and K^+^ efflux. The decrease of intracellular K^+^ under a critical threshold definitively impairs the volume regulatory systems and leads to a sudden increase of hepatocytes volume, with osmotic lysis and death of hepatocytes [35].

Interestingly hypoxic preconditioning and A2aR activation prevent the irreversible Na^+^ increase that precedes hypoxic hepatocytes damage. As shown in Figure 1, A2aR stimulation allows the maintenance of intracellular pH and prevents the activation of the Na^+^-dependent systems of pH regulation [19, 36]. Such effect is p38 MAPK- and PI3K-dependent and is due to the activation and translocation to the plasma membrane of the vacuolar ATPase (V-ATPase). V-ATPase acts as alternative pH buffering system and allows proton extrusion avoiding the irreversible Na^+^ accumulation that precipitates hypoxic hepatocytes death [36, 37].

The protective effects of A2aR stimulation can be either immediate (early preconditioning) or delayed (late preconditioning). Early preconditioning allows hepatocytes to respond to a pathogenic stress that immediately follows the preconditioning stimulus and involves the activation of constitutive molecular systems. Late preconditioning is, instead, able to increase hepatocytes resistance to hypoxia up to 24 hours after the preconditioning stimulus and involves DNA transcription and* de novo* protein synthesis. Hypoxia-inducible factor 1 (HIF-1) is the main regulator of tissue adaptation to oxygen deprivation [38] and it is found to be increased in human transplanted livers exposed to IP [39]. Consistently we found that late hypoxic preconditioning of primary cultured hepatocytes is mediated by an A2aR-dependent nonhypoxic HIF-1 activation and the consequent production of its target protein carbonic anhydrase IX (CAIX) [34]. As shown in Figure 1, A2aR induces a PI3K- and PKC-dependent nuclear translocation, DNA binding, and activation of the nuclear transcription factor HIF-1. In turn, HIF-1 induces the expression of CAIX that converts CO_2_ into bicarbonate in the extracellular milieu. Bicarbonate then is transported into the hepatocytes through the Cl^−^/CO_3_ exchanger and neutralizes the intracellular acids, thus maintaining the physiological cytosolic pH and preventing Na^+^ accumulation [40].

## 5. Adenosine A2a Receptor Activation Protects Hepatocytes Lipotoxicity

The shortage of organs for liver transplantation has led to expansion of the criteria for the acceptance of marginal donors, including the use of steatotic grafts [41]. Steatosis is characterized by accumulation of excess fat, that is, when the lipid content in cell exceeds 5% of lipid of total liver weight. Steatosis is the most frequent hepatic lesion in western countries with prevalence in the general population ranging from 3% to 15% but reaching up to 70% among overweight individuals [41, 42]. Importantly the presence of fatty infiltration dramatically reduces the tolerance of the liver to I/R injury in experimental models [43] increasing pathological consequences of I/R upon human liver surgery. Indeed clinical meta-analysis shows that patients with steatosis have an up to twofold increased risk of postoperative complications, and those with excessive steatosis have an almost threefold increased risk of death [44]. Several factors such as an increase of oxidative stress, mitochondrial alterations, and ATP depletion can participate in the decreased tolerance of steatotic liver to I/R injury compared with normal livers [45–48]. The accumulating lines of evidence on the phenomenon known as lipotoxicity [49] indicate that the hepatotoxic effects of free fatty acids may represent further attractive mediators of this process. The pathophysiological picture of steatosis is, in fact, characterized by an increase of circulating nonesterified free fatty acids and their metabolites [50] which have been shown to induce hepatic cell apoptosis through JNK activation [45].

The application of IP to fatty livers has demonstrated interesting results. IP, in fact, almost halved transaminase release and the histological evidence of liver cell death showing a greater efficacy of IP in steatotic liver compared to normal liver [48]. The mechanisms responsible for these beneficial effects are, however, unclear.

**Figure 2:**
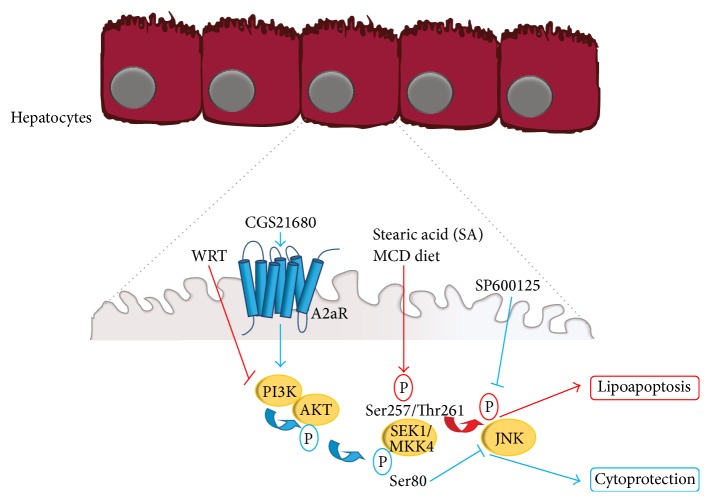
Molecular mechanisms involved in the lipotoxic effects of free fatty acids and in their protection upon A2aR stimulation. Stearic acid (SA) induces primary rat hepatocyte apoptosis by activating JNK-1/2 through the stimulation of MKK4/SEK1. A2aR activation prevents apoptosis by a PI3K/Akt-dependent inhibition of MKK4/SEK1. (See also text and [51, 67].)

In recent studies, we evaluated the capacity of A2aR stimulation to prevent lipoapoptosis of primary rat hepatocytes and to inhibit the development of nonalcoholic steatohepatitis in rat fed with MCD (methionine choline-deficient) diet [51]. The treatment of primary rat hepatocytes with the free fatty acid, stearic acid (SA), promoted apoptosis by inducing MKK4 (mitogen activated protein kinase kinase-4)/SEK1 (stress-activated protein kinase/extracellular-signal regulated kinase kinase-1) and JNK-1/2 (c-Jun N-terminal kinase-1/2) activation (Figure 2). The pharmacological A2aR stimulation prevented JNK-1/2 activation by a PI3K/Akt-mediated block of MKK4/SEK1 and also protected lipoapoptosis* in vitro* (Figure 2) and the progression of steatosis to steatohepatitis* in vivo* [45]. These findings may have multiple implications. First, A2aR activation is able to exert separate protective effects against lipotoxicity associated steatosis and against I/R. This may account for additive protective action of A2aR activation and for the increased efficacy of IP in preventing I/R injury in fatty liver (researches are in progress to investigate this point). In addition, the capacity of a molecular inductor of IP to protect against hepatic insults also different from I/R injury potentially broadens the field of clinical application of IP. The activation of IP by pharmacological stimulation of one or some of its mediators would allow, in fact, its employment in all the clinical settings where the chirurgical IP is not applicable.

## 6. Proteome Reveals Protection Mechanisms in Preconditioned Hepatocytes and LSECs

An important approach to identify new protein mediators of liver preconditioning is the use of the proteomic analysis. In a recent research we evaluated the proteomic patterns of primary hepatocytes and sinusoidal endothelial cells (LSECs) isolated from mice liver following I/R with or without pretreatment with the A2aR agonist CGS21680 [52]. Hepatocytes and LSECs are the main targets of I/R injury and of the beneficial effects of IP. In comparison to hepatocytes, the knowledge of the molecular mechanisms responsible for the effects of I/R and IP on LSECs is very limited [53]. LSECs, however, have been demonstrated to be largely sensitive to ischemic preservation and I/R [54]. Early studies showed that cultured LSECs exposed to hypoxia-reoxygenation produce high level of oxidative species that can lead to LSECs damage [54]. More recently, ischemic preservation of LSECs demonstrates the downregulation of the transcription factor Kruppel-like factor 2 (KLF2) [55] that is involved in the induction of a number of protective genes including the transcription factor Nrf2 that controls the expression of several antioxidant enzymes such as NAD(P)H dehydrogenase, quinone 1 (NQO1), glutathione peroxidase (GPX), or heme-oxygenase 1 (HO-1) [56]. Consistently recent reports show that remote or intestinal preconditioning prevents hepatic I/R injury via HO-1 mediated mechanisms [57, 58]. In addition the microcirculatory disturbances are a hallmark of hepatic I/R injury [59] and IP application was demonstrated to prevent both LSECs damage and microcirculatory alteration [60, 61].

The employment of proteomic analysis allowed us to evidence profound changes of hepatocytes and LSECs proteome, providing new insights into some critical aspects of I/R injury and IP-induced hepatoprotection. In particular, we observed the modulation of several proteins involved in response to apoptosis and in regeneration and cell signalling and, more importantly, we found major modifications in enzymes involved in oxidative stress protection and energy production, two fundamental processes affected by I/R and IP.

Previous studies clearly showed an increased production of oxidative species during hepatic I/R as well as the capacity of IP to prevent such damaging process [1, 4, 5, 56, 62]. Consistently we evidenced the modulation of several proteins involved in cell response to oxidative stress such as catalase, glutathione transferases GSTP1, GSTP2, and GSTM1, and peroxiredoxin 6. Notably we observed that I/R depressed the antioxidant enzymes content in LSECs exclusively, while A2aR stimulation generally increased the antioxidant defences in both LSECs and hepatocytes. These findings provide a rational base to the greater susceptibility of LSECs to oxidative stress [54] and are consistent with the possible downmodulation of Nrf2 [56]. Our observations indicate, moreover, that the ability of preconditioning to protect against I/R-induced oxidative stress can be explained by an increased antioxidant enzymes expression.

Another critical process is the decrease of ATP content in liver exposed to I/R and its prevention upon preexposure to IP [1–5]. Consistently the proteomic analysis shed light on large modification of enzymes involved in the transport and catabolism of metabolites necessary for energy production. We have observed that I/R induces in hepatocytes and LSECs a decrease of enzymes involved in carbohydrate and lipid catabolism. On the contrary, A2aR stimulation not only rescued the enzymes downregulated by I/R, but even increased enzymes associated with carbohydrate and aminoacids and lipid supply and catabolism. In the specific case of the glycolytic metabolism we found that almost the entire pathway was upregulated in both hepatocytes and LSECs.

The severe ATP depletion of liver tissue exposed to I/R is generally ascribed to the lack of O_2_ and glycolytic substrates supply consequent to blood interruption during ischemia [1–3]. Our results indicate that the decrease in the efficiency of the pathways involved in the anaerobic ATP production can significantly exacerbate this process. On the other hand, the rescue or increase of the same pathways by A2aR stimulation can explain the maintenance of the ATP content of preconditioned liver. Another critical aspect is the inability of I/R-injured liver to recover aerobic ATP production at blood flow reestablishment during reperfusion and, on the other hand, the ability of IP to prevent such alteration [1–3]. We observed that I/R inhibited in both hepatocytes and LSECs ATP synthesis downmodulating the regulatory subunit B of ATP synthase and also affecting the catalytic subunit A that is essential for completion of the synthase activity. On the other hand, CGS21680 upregulated in hepatocytes and LSECs both ATPA and ATPB and also, in LSECs, the additional catalytic subunit D (ATPH5). Furthermore, in both cells, CGS21680 increased the electron transfer flavoprotein subunit alpha (ETFA), active in oxidative phosphorylation, and, in hepatocytes, S2542, a carrier mediating the transport of CoA in mitochondria that will then enter in the Krebs cycle to produce ATP. This indicated that I/R, by decreasing the enzymes of the mitochondrial metabolism, affects the capacity to synthesize ATP also in presence of O_2_ and that A2aR activation restores this process by rescuing or even increasing these enzymes.

**Figure 3:**
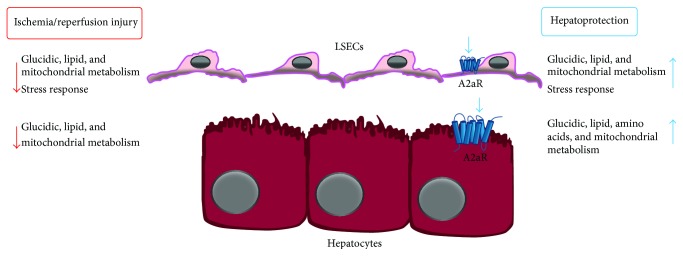
Main proteomic changes of hepatocytes and LSECs upon A2aR stimulation and/or hepatic ischemia/reperfusion. Mice liver exposure to ischemia/reperfusion (I/R) downmodulates proteins associated with glucidic, lipid, and mitochondrial metabolism in hepatocytes and LSECs and, specifically in LSECs, with stress response. These proteins or proteins associated with the same processes are restored or upregulated in both hepatocytes and LSECs, upon A2aR stimulation after mice* in vivo* treatment with the A2aR agonist CGS21680. (See also text and [52].)

Altogether, these results showed that hepatic cells isolated from liver exposed to I/R develop a “pathological phenotype” characterized by a decrease of the metabolic enzymes involved in the aerobic and anaerobic ATP production and, in the specific case of LSECs, an additional decrement of antioxidant defences (Figure 3). On the contrary, A2aR stimulation induces the expression of a “protected phenotype” characterized by an enhancement of enzymes necessary for energy production and ROS detoxification (Figure 3). This gives a sort of metabolic and antioxidant advantage to preconditioned compared to nonpreconditioned cells and can account for the increased resistance to death of preconditioned hepatic tissue during I/R exposure.

## 7. Clues for Novel Pharmacological Approaches to Minimize Ischemia/Reperfusion in Patients

The analysis of the molecular changes induced by A2aR stimulation suggests novel potential pharmacological strategies to be applied in human hepatic surgery. First, the findings of the multiple mechanisms of liver cell protection induced by A2aR activation strongly enforce the idea to translate A2aR agonists to the clinical practise as hepatoprotective tool. In addition to the chemical A2aR agonists such as CGS21680, apadenoson (ALT-146), and ATL-313 largely employed in the preclinical models (see [63] for review), pharmacological agents leading to A2aR activation are already available for clinical purpose in humans. For example, the compound known as regadenoson (CVT-3146) is already approved by the U.S. Food and Drug Administration and it is in use as coronary vasodilator [64, 65].

Additionally, the molecular identification of pleiotropic effects of A2aR stimulation implicates the possibility to intensify these beneficial effects by a concomitant stimulation of their mediators. Moreover, in relation to the needed clinical setting, it might be of interest to achieve a focused stimulation of specific protective signals. For example, in case of short surgical hepatic interventions, it might be favourable to stimulate the protective network of early preconditioning. The choice could be then a simultaneous treatment with A2aR agonists and DGK and PTEN inhibitors in order to sustain the A2aR-induced repression of the negative regulators of PKC and PI3K that are activated within the first hour after stimulation of A2aR. In case of prolonged interventions, like those necessary for major liver surgery and transplantation, the cocktail treatment could additionally include items able to sustain HIF activation such prolyl hydroxylase inhibitors that appear to be well tolerated in patients [66]. Critical would be also the exploitation of antioxidant and metabolic advantages of preconditioned liver cells. In particular, the increased antioxidant enzymatic efficiency of A2aR preconditioned liver cells could be improved by the inclusion in liver graft conservation solutions of natural or synthetic antioxidants [67]. On the other hand, the increased metabolic activities of preconditioned liver cells can take a further advantage by the supplementation with energy-linked metabolites to sustain the glucidic, aminoacids, and lipid catabolism and thus anaerobic and aerobic ATP production.

## Abbreviations

IP: Ischemic preconditioning
I/R: Ischemia/reperfusion
A2aR: Adenosine A2a receptor
ROS: Reactive oxygen species
PKA: Protein kinase A
PI3K: Phosphatidylinositol-3-kinase
PKC: Protein kinase C
DGK: Diacylglycerol kinases
DAG: Diacylglycerol
PTEN: Phosphatase and tensin homologue deleted from chromosome 10
V-ATPase: Vacuolar ATPase
HIF-1: Hypoxia-inducible factor 1
CAIX: Carbonic anhydrase IX
SA: Stearic acid
MKK4: Mitogen activated protein kinase kinase-4
SEK1: Stress-activated protein kinase/extracellular-signal regulated kinase kinase-1
JNK 1/2: c-Jun N-terminal kinase-1/2
LSECs: Sinusoidal endothelial cells
KLF2: Kruppel-like factor 2
GST: Glutathione transferase
NQO1: NAD(P)H dehydrogenase quinone 1
GPX: Glutathione peroxidase
HO-1: Heme-oxygenase 1
ETFA: Electron transfer flavoprotein subunit alpha.
